# Selective depletion of naïve T cells by targeting CD45RA

**DOI:** 10.3389/fonc.2022.1009143

**Published:** 2023-01-27

**Authors:** Swati Naik, Brandon M. Triplett

**Affiliations:** Department of Bone Marrow Transplantation and Cellular Therapy, St. Jude Children’s Research Hospital, Memphis, TN, United States

**Keywords:** T-cell depletion, CD45RA depletion, memory T cells, Hematopoietic Cell Transplantation, naive T cell depletion

## Introduction

Allogeneic hematopoietic cell transplantation (HCT) can provide a curative option for patients with high-risk hematological malignancies and several non-malignant hematological disorders (1). In addition to hematopoietic progenitor cells, which can replace and repopulate the host hematopoietic system, the donor graft comprises a host of immune effectors that are increasingly being recognized as key mediators of post-transplant outcomes (2–4).

The most important and well-studied immune effectors are donor T cells. They play a crucial role in the transplant process, and the extent to which the sensitive balance of their effector functions is maintained is a primary determinant of clinical outcomes (5, 6). Donor T cells can facilitate engraftment, accelerate immune reconstitution, minimize the risk of infectious complications, and mediate a graft-versus-leukemia (GVL) effect that can decrease the risk of relapse. However, these cells can also cause graft-versus-host disease (GVHD) to develop, and the risk of GVHD increases with the degree of HLA-mismatch between the transplant donor and the recipient. Transplants from matched sibling donors (MSDs) have the lowest risk of causing GVHD, followed by those from HLA-matched unrelated donors (MUDs), whereas the highest risk of GVHD is associated with HLA-mismatched unrelated donors (MMUDs) and haploidentical (haplo) donors (7, 8).

Several strategies have been explored to reduce the risk of GVHD (9). One such strategy involves graft engineering, whereby T cells in the donor graft are depleted ex vivo (10). Early approaches to ex vivo T-cell depletion involved pan–T-cell depletion techniques such as negative depletion of CD3^+^ T cells or positive selection of CD34^+^ cells, both of which lead to profound depletion of all donor T-cell subsets. These approaches were generally successful, but recipients were plagued by severely delayed immune reconstitution and consequently high rates of serious viral infections and disease relapse (11, 12).

As a result of improved understanding of T-cell development and biology, it is now known that specific subsets of T cells may contribute to the development of GVHD (5, 13). Current efforts are, therefore, focused on highly nuanced, selective forms of T-cell depletion to optimize the immunologic composition of donor grafts (9, 11). One such form of selective T-cell depletion involves depleting naïve T cells from the donor graft. Broadly speaking, T cells can be divided into naïve T cells that have not yet encountered their cognate antigen and memory T cells that have previously responded to their specific antigen (14). The roles of naïve and memory T cells in the development of GVHD have been extensively studied in the preclinical setting *in vitro* and in murine models, and this has led to the translation of naïve T-cell depletion to clinical trials (15). The results of these preclinical experiments and the subsequent translational work are reviewed below.

## Preclinical data

CD45, also called the leukocyte common antigen, is a cell surface glycoprotein with tyrosine phosphatase activity, that is expressed on nearly all hematopoietic cells in various isoforms related to their stage of development and activation (16). One of the isoforms is CD45RA, which identifies naïve T cells (17). Mature T-cells are considered to be immunologically naive until they encounter the specific peptide in the context of an human leukocyte antigen (HLA) molecule that their receptor recognizes (18). Once antigen recognition occurs, the cells receive a proliferative signal that leads to marked, antigen-specific T-cell expansion and an inflammatory response. Although many of these cells undergo apoptosis after the initial response, others are rescued from immune retraction and persist as memory T cells, most of which then become CD45RA negative (19). Memory T cells can respond rapidly to an antigen specific rechallenge (20), and persist in the circulation long-term, as a diverse cell pool that includes central memory T-cell (T_CM_) and effector memory T-cell (T_EM_) subsets, among others (21).

Anderson and colleagues were the first to ask whether naïve T cells or memory T cells were implicated in GVHD development (22). Using a CD4^+^-dependent, MHC-matched, minor histocompatibility (H) antigen–mismatched chronic GVHD model, they asked whether effector memory CD62L^−^CD44^+^CD4^+^ T cells were more or less likely to induce GVHD than were unfractionated or naïve CD62L^+^CD44^−^CD4^+^ T cells. Impressively, they found that effector memory CD4^+^ T cells induced neither clinical nor histologic GVHD. To ensure that the lack of GVHD was not related to an increase in CD4^+^CD25^+^ regulatory T cells (Tregs), they performed additional experiments in which they depleted Tregs before a T-cell infusion and observed that the Treg-depleted naïve T cells still caused GVHD whereas the Treg-depleted memory T cells did not. Furthermore, memory CD4^+^ T cells engrafted and could mount strong proliferative recall responses when challenged *in vivo*, highlighting the potential to retain responses against pathogens and tumors. These findings have been confirmed in several preclinical murine models of GVHD (23–27).

Further work consistently showed that T_EM_ cells were greatly impaired in their ability to induce GVHD (22–25, 28–30) and that they were never associated with GVHD development. The capacity of T_CM_ cells to induce GVHD was less well defined, and studies yielded conflicting data. Zheng et al. clearly demonstrated that T_CM_ cells did cause GVHD but that this was a clinically milder form of GVHD when compared to that caused by naïve T cells (31). However, in contrast to their differing ability to induce GVHD, CD8^+^ T_CM_ cells and naïve T cells were comparable in their ability to mediate a GVL effect.

Several groups have attempted to understand the differences in the properties of these T-cell subsets in order to explain the mechanisms of GVHD induction or lack thereof. Anderson et al. investigated the role of the T-cell receptor (TCR) repertoire and showed that a reduction in the alloreactive T-cell repertoire among T_EM_ cells did not by itself explain the reduced capacity of such cells to cause GVHD (26). The authors considered that, as trafficking patterns alone were insufficient to explain differences in the ability of naïve T cells and T_EM_ cells to cause GVHD (25, 28, 32), other inherent biologic differences between naïve T cells and T_EM_ cells might influence the outcomes. These differences could include the reduced ability of T_EM_ cells to clonally expand (33, 34), their reduced ability to survive (35), and their distinct effector functions (36) when compared to naïve T cells. Further work is required to elucidate the specific nature of the mechanistic differences involved.

Bleakley and colleagues also evaluated human T-cell responses to minor (H) antigens *in vitro* (37). They showed that naïve T cells had a frequency of minor (H) antigen–specific T cells that was up to 20 times higher than that in the memory T-cell population. In addition, upon further expansion, the T-cell lines derived from naïve T cells remained cytotoxic, whereas those derived from memory T cells lost their response to minor H antigens. These data suggested that depletion of naïve T cells was also likely to reduce minor H antigen–specific T cells thereby reducing the risk of GVHD, and that any minor H antigen T cells derived from memory T cells would be unlikely to cause severe GVHD.

Importantly, the elimination of naïve T cells from an infused cell product would not significantly affect responses to infection, as memory T cells can transfer immunity to pathogens and retain anti-leukemic properties (22, 27, 29, 31). Furthermore, it is the CD45RA^−^ memory cells that are responsible for aiding B-cell differentiation and antibody production.

In summary, these findings show that naïve T cells can cause severe GVHD, that T_CM_ cells induce milder GVHD, and that T_EM_ cells do not cause GVHD at all (22–25, 29, 31). Therefore, depleting naïve T cells in the infused product could reduce the rates of GVHD in patients without compromising the response to pathogens or leukemia-specific antigens.

## Engineering the graft

These observations led to the hypothesis that using naïve or CD45RA^+^-depleted grafts while preserving memory T cells with specificity for viral and leukemia antigens would be associated with low rates of GVHD. Accordingly, procedures to engineer such grafts were devised.

As shown by Bleakley et al, CD45RA^+^ is not subject to proteolysis by metalloproteases released during G-CSF mobilization of peripheral blood stem cells and is therefore ideal for targeted depletion unlike other markers such as CD62L, which are also selectively expressed on naïve T-cells (38).

Several groups including ours have shown that it is feasible to effectively deplete naïve T-cells using immunomagnetic CD45RA^+^ depletion (38–41). Briefly, following apheresis of G-CSF mobilized peripheral blood, the cells are incubated with a GMP-grade CD45RA microbead reagent to label them. This is followed by washing of the cells to remove unbound beads. The CD45RA labeled cells are then applied to the CliniMACS (Miltenyi Biotec GmbH, Bergisch Gladbach, Germany) device and the CD45RA^+^ cells are removed using a depletion program. Flow cytometric analysis before and after depletion is performed to assay the subsets. Using this technique, depletion of CD45RA^+^ cells leads to a >4 log depletion of naïve T-cells. In addition, as there is expression of CD45RA on other cells of the hematopoietic lineage, there is also a 2-4 log depletion of B-cells and 1-3 log depletion of NK cells. Importantly however, multifunctional CD4+ and CD8+ T cells capable of specifically responding to viral antigens are retained in CD45RA^+^-depleted grafts suggesting that protective immunity could be transferred (38) and alloreactive CD8+ T-cells are significantly reduced suggesting that the use of a CD45RA^+^ depleted allogeneic graft could be associated with a lower risk of inducing GVHD (39).

## Clinical data

CD45RA^+^-depleted cells have been used in the transplant setting as progenitor cell grafts and as unstimulated lymphocyte infusions after transplant. The published clinical results are summarized below:

## Progenitor cell grafts

CD45RA^+^-depleted progenitor grafts have been used in the context of MSD, MUD, MMUD, and haplo donor transplants **Table 1A**.

**Table 1A:** Clinical trials and outcomes after transplant with CD45RA-depleted progenitor cell grafts.

Author, Clinical Site, Ct.gov Id (If Available)	Diagnosis, Patient Numbers	Donor Type	Conditioning	GVHD Prophylaxis	Graft Composition Median (range)	Survival	Cumulative Incidence of Acute GVHD	Cumulative Incidence of Chronic GVHD
Bleakley et al. (42)	Adults with acute leukemia, MDS (n = 35)	MSD (n = 35)	Myeloablative conditioning	Tacro (n = 35)	CD34^+^ cells/kg: 7.4 × 10^6^ (range: 5.1 × 10^6^ to 19.9 × 10^6^) CD3^+^ T cells/kg: 10x 10^6^ (range: 1.6 × 10^6^ to 10.2 × 10^6^) CD3^+^ CD45RA^+^ CD45RO^-^cells/kg: 0.36 × 10^4^ (range: 0.05 × 10^4^ to 7.46 × 10^4^)	2-year OS: 78% (95% CI: 59%– 89%) 2-year DFS: 70% (95% CI: 51%–83%)	Grade II–IV: 66% (95% CI: 41%–74%) Grade III: 9% (95% CI: 0%–18%) Grade IV: 0%	At 2 years: 9% (95% CI: 0%–19%)
Bleakley et al. (43), multicenter, (NCT01858740, NCT02220985)	Children and adults with acute leukemia, MDS (n = 138)	MSD (n = 84) MUD (n = 54)	High-intensity regimen (n = 100) Intermediate-intensity regimens (n = 38)	Tacro (n = 41) Tacro/MTX (n = 59) Tacro/MMF (n = 38)	CD34^+^ cells/kg: 8.4 – 10^6^ (range: 3.8 × 10^6^ to 20 × 10^6^) CD3^+^ T cells/kg: 10 × 10^6^ (range: 1.6 × 10^6^ to 10.2 × 10^6^) CD3^+^ CD45RA^+^ CD45RO^-^ cells/kg: 0.25 × 10^4^ (range: 0.03 × 10^4^ to 7.46 × 10^4^)	3-year OS: 77% (95% CI: 71%–85%) 3-year RFS: 69% (95% CI: 61%–77%)	Grade II: 71% (95% CI: 64%–79%) Grade III: 4% (95% CI: 1%–8%) Grade IV: 0%	At 3 years: Mild: 6% (95%CI: 2%–10%) Moderate: 1% (95%CI: 0%–2%) Severe: 0%
Touzot et al. (44), France	Children with combined immune deficiency (n = 5)	MMUD (n = 5)	Myeloablative conditioning regimen		CD34^+^ cells/kg: 11 × 10^6^/kg (range: 3 × 10^6^ to 18 × 10^6^/kg) CD3^+^ T cells/kg: 8.95 × 10^7^ (range: 3.87 × 10^7^ to 14.8 × 10^7^).	4 of 5 patients alive	1 patient with grade I skin GVHD	–
Gasior Kabat et al. (45), Spain	Children with severe aplastic anemia (n = 4) Congenital amegakaryocytic thrombocytopenia (n = 1)	Haplo (n = 4) MMUD (n = 1)	Submyeloablative conditioning regimen	CSA and/or MMF and/or steroids	Mean cells/kg in CD34^+^ fraction: 6.58 × 10^6^ (range: 5 × 10^6^ to 7.73 × 10^6^) Mean cells/kg in CD45RA-depleted fraction: 3.84 × 10^7^ (range: 2.8 × 10^7^ to 5 × 10^7^)	3 of 5 patients alive	Grade II–IV: 4 patients	1 patient
Naik et al. (46), St. Jude (NCT0180761)	Children with hematologic malignancies (n = 72)	Haplo (n = 72) CR1: n = 25; CR2+: n = 28; refractory disease: n =19	Submyeloablative conditioning (n = 72)	MMF (n = 61) and/or sirolimus (n = 8)	i) Day 0: CD34-selected graft. CD34^+^ cells/kg: 9.85 × 10^6^ (range: 1.96 × 10^6^ to 44.64 × 10^6^) ii) Day +1: CD45RA-depleted graft. CD34^+^ cells/kg: 5.82 × 10^6^ (range: 0.58 × 10^6^ to 39.43 × 10^6^) CD3^+^ T cells/kg: 60.1 × 10^6^ (range: 16.08 × 10^6^ to 528.43 × 10^6^) CD3^+^CD45RA^+^ cells/kg: <0.001 × 10^6^ (range: 0 to 0.2 × 10^6^) iii) Day +6: NK cells/kg: 11.7 × 10^6^ (range: 1.65 × 10^6^ to 99.2 × 10^6^)	3-year OS: 68.9% (95% CI: 58.9%–80.6%) 3-year LFS: 62.2% (95% CI: 51.9%–74.6%) CR1: 88% (95% CI: 76.1%–100%). CR2+: 70.8% (95% CI: 54.8%–91.6%); refractory disease: 21.1% (95% CI: 8.81%–50.3%)	Grade II–IV: 36.1% (95% CI: 25.1%–47.2%) Grade III or IV: 29.2% (95% CI: 19.1%–39.9%)	At 3 years: 20.8% (95% CI: 12.3%–30.9%)
Sisinni et al. (47), Spain	Children with acute leukemias (n = 25)	Haplo (n = 25)	Submyeloablative conditioning (n = 72)	CSA (n = 3), CSA+MTX (n = 1), MMF (n = 21)	CD34^+^ cells/kg: 6.29 × 10^6^ (range: 4.04 × 10^6^ to 18.1 × 10^6^) CD45RA^+^ cells/kg: 0.6 × 10^4^ (range: 0.2 × 10^4^ to 1 × 10^4^)	OS at 30 months: 58%	Grade II–IV: 39% Grade III or IV: 33%	At 30 months: 22%
Gasior et al. (48), Spain	Children with hematologic malignancies (n = 17) Severe aplastic anemia (n = 1)	Haplo (n = 16) MSD (n = 2)	Submyeloablative conditioning	MMF (n = 18)	CD34^+^ cells/kg: 6.5 × 10^6^ (range: 5 × 10^6^ to 11.2 × 10^6^) CD3^+^CD45RA^+^ cells/kg: 3.6 × 10^4^ (range: 0 to 23 × 10^4^) Day +7: NK cells/kg: 12.6 × 10^6^ (range: 3.9 × 10^6^ to 100 × 10^6^)	2-year OS: 87.2% (95% CI: 78.6%–95.8%) 2-year EFS: 67.3% (95% CI: 53.1%–81.5%) 2-year GRFS: 64% (95% CI: 50.5%–78.1%)	At day +180: Gr III or IV: 34.8% (95% CI: 21.8%–47.8%)	At 1 year: 23.1% (95% CI: 11.4%–34.1%)

GVHD, graft-versus-host disease; MDS, myelodysplastic syndrome; MSD, matched sibling donor; MUD, matched unrelated donor; MMUD, mismatched unrelated donor; Haplo, haploidentical donor; CR, complete remission; Tacro, tacrolimus; MTX, methotrexate; MMF, mycophenolate mofetil; CSA, cyclosporine A; OS, overall survival; DFS, disease-free survival; LFS, leukemia-free survival; EFS, event-free survival; GRFS, GVHD-free, relapse-free survival.

## Matched sibling donors

The first-in-human trial of naïve T-cell depleted grafts was reported by Bleakley et al. (42). They performed a two-step depletion of mobilized grafts, in which CD34^+^ selection was followed by depletion of CD45RA^+^ cells from the CD34^+^-negative fraction. They demonstrated that it was feasible to engineer grafts so that they retained CD34^+^ progenitor cells and memory T cells while being effectively depleted of naïve CD45RA^+^ T cells. Indeed, their strategy achieved a profound depletion of naïve T cells to less than 7.5 × 10^4^ cells/kg. In their study, 35 patients with high-risk acute leukemia received these CD45RA^+^-depleted grafts from MSDs after myeloablative conditioning. No serotherapy was used, and GVHD prophylaxis consisted of single-agent tacrolimus. Engraftment was a primary safety endpoint because T-cell depletion has historically been associated with high rates of graft failure, and engraftment was successful in all recipients, although one of 35 recipients developed secondary graft failure at day 260 despite 100% donor chimerism. Acute GVHD (aGVHD) was a second primary endpoint, and although its incidence was not reduced by this procedure, it was mostly limited to the upper gastrointestinal tract without skin or liver involvement. Importantly, there were no cases of steroid-refractory aGVHD in transplant recipients; in most patients, aGVHD resolved rapidly and completely within 7 days of steroid initiation. However, the most notable finding was the remarkably low rate of chronic GVHD (cGVHD), which was comparable to that reported after pan–T-cell depletion and was significantly lower than that reported with other forms of T-cell–replete MSD transplants.

Immune reconstitution was rapid and robust when compared to that observed with other methods of T-cell depletion and was accompanied by the transfer of protective virus-specific immunity. There were no cases of Epstein–Barr virus–associated post-transplant lymphoproliferative disease (EBV-PTLD), and the authors demonstrated expansion of cytomegalovirus (CMV)-specific CD8^+^ T cells in patients in response to CMV reactivation, before the emergence of T-cell receptor excision circles (TRECs), which is consistent with the transfer of functional anti-CMV cellular immunity from the infused graft. This translated to low rates of non-relapse mortality (NRM). In addition, despite the reduction in cGVHD, there was no adverse impact on disease-free survival (DFS) and rates of relapse. The 2-year DFS of 70% compares favorably to the 2-year DFS rates seen after pan–T cell–depleted or T-cell–replete MSD transplants. Furthermore, the relapse rate of 21% at 2 years in this cohort compares favorably to published relapse incidences for patients with acute leukemia using different HCT approaches, implying that the GVL effect of memory T cells is probably not abrogated by this approach

## Unrelated donors

Bleakley and colleagues also recently published data from three phase II trials of T-naïve–depleted peripheral blood stem cell (PBSC) grafts, including the outcomes in 138 recipients who had underlying acute leukemia or myelodysplastic syndrome (MDS) (43). The first trial (NCT00914940) included only patients with MSD who underwent myeloablative conditioning. The results for the first 35 patients were previously published and are described above. The other two trials (NCT01858740 and NCT02220985) included both MSD and MUD transplants. Conditioning consisted of a high-intensity myeloablative regimen in one trial (NCT01858740) and both high-intensity and intermediate-intensity regimens in the other (NCT02220985). Here again, no serotherapy was used and GVHD prophylaxis consisted of either tacrolimus plus methotrexate (Tac/MTX) for the high-intensity regimen or tacrolimus plus mycophenolate mofetil (Tac/MMF) for the intermediate-intensity regimen. The same two-step graft engineering approach described above was used, and the graft composition across the trials was similar, highlighting the feasibility and general applicability of this approach. Engraftment occurred in all evaluable patients, but six patients died during the first 100 days, before neutrophil (n = 1) or platelet engraftment (n = 6). The cumulative incidences of grade III and grade IV aGVHD were very low, regardless of the donor source or the conditioning intensity. Most of the aGVHD that developed was stage 1 (upper) gastrointestinal tract GVHD that was responsive to corticosteroids, and in all but two cases, it was treated with steroids alone without the need for a second agent. Once again, the rates of cGVHD were remarkably low, much lower than those seen with T-cell–replete MSD or MUD grafts. Importantly, there was no increase in NRM; this was probably related to the low rates of serious infectious complications and the absence of EBV-PTLD. Furthermore, despite the reduction in cGVHD, there was no increase in the rates of relapse relative to those with standard T-cell–replete transplants. Of note, in this dataset, aGVHD development was associated with a decreased risk of relapse and death, suggesting a connection to GVL and an alloimmune mechanism. The disconnect between the occurrence of aGVHD and cGVHD also implies differences in the biological properties of naïve and memory T cells in mediating these responses. Overall, the low incidence of cGVHD with no increase in the rates of relapse or NRM was associated with impressive rates of overall survival (OS), relapse-free survival (RFS), and GVHD-free, relapse-free survival (GRFS).

cGVHD is associated with long-term morbidity and mortality and has a detrimental effect on the quality of life of survivors. If these low rates of cGVHD can be obtained without increasing the rates of relapse, this strategy has the potential to substantially change the landscape of transplantation. Furthermore, these studies also confirm that naïve T-cell depletion is feasible in the setting of MUD transplants and that lower-intensity regimens are adequate to ensure engraftment.

As Bleakley et al. note, randomized trials will be needed to confirm these findings and to ensure that there is no negative impact on relapse and on composite endpoints, such as GRFS, in recipients of naïve T-cell–depleted HCTs, compared to patients treated with standard HCT or using other GVHD-reduction strategies. The same group of investigators has, therefore, initiated two phase II randomized control trials: NCT03970096, which compares naïve T-cell–depleted PBSC transplantation to T-cell–replete PBSC transplantation with Tac/MTX or with PTCy, and NCT03779854, which is a Pediatric Transplantation and Cellular Therapy Consortium trial comparing naïve T-cell–depleted PBSC transplantation to T-cell–replete bone marrow transplantation with Tac/MTX.

Touzot and colleagues used the approach of naïve T-cell depletion in the MMUD setting in five patients with combined immune deficiencies (CIDs) (44). Naïve T cells were rigorously depleted from the graft, confirming the reliability of this approach. All patients received myeloablative conditioning incorporating ATG and GVHD prophylaxis consisting of cyclosporine and MMF. Engraftment occurred in four of the five patients; the one patient who did not engraft received a low dose of CD34^+^ cells in their graft. Here again, immune reconstitution was rapid, there was clearance of all viruses detected before HCT, and there were no new clinically relevant viral infections. The investigators noted expansion of CD8^+^ CMV-specific T cells that coincided with the clearance of CMV viremia. There were no cases of grade III or IV GVHD. Taken together, these data suggest that this approach would be effective for patients with CIDs or other immune deficiencies who are at high risk for GVHD and infection when receiving transplants from MMUDs.

## Haploidentical donors

At our institution, we have used CD45RA^+^-depleted progenitor grafts derived from haplo donors. The results obtained with the CD45RA^+^ depletion strategy compare very favorably with those obtained with the other methods of T-cell depletion previously employed at our institution. We previously reported the outcomes of 143 pediatric and young adult patients with hematologic malignancies who received a first allogeneic HCT on one of six consecutive ex vivo T-cell–depleted haplo transplant protocols over the past 15 years at our institution, including the first 50 patients treated on the CD45RA^+^-depleted haplo transplant study described below (49). Our data demonstrated increased 3-year OS and EFS in non-chemo refractory recipients who received CD45RA^+^-depleted grafts, as compared with historical T-cell–depleted haplo transplant cohorts (OS: 78.9% *vs* 46.7%, *P* = 0.004; EFS: 77.7% *vs*. 42.7%; *P* = 0.003). This improvement was primarily due to a reduction in NRM. Historical haplo transplant cohorts had increased rates of aGVHD and cGVHD when the infused T-cell dose was >0.1 million/kg. In comparison, 1000-fold higher T-cell doses were infused without a significant increase in GVHD when CD45RA^+^ depletion was used. However, a skewing towards more severe aGVHD was noted in these patients. Although preclinical studies and other reported clinical studies have shown lower rates of GVHD with CD45RA^+^-depleted T cells, these results may indicate cross-reactivity of pathogen-specific CD45RA^+^-depleted T cells against allo antigens. Importantly, although the rates of grade II–IV GVHD were higher (cumulative incidence of 32%) than reported in other studies involving CD45RA^+^ depletion, the 2-year OS and cumulative incidence of NRM calculated from time of onset of severe grade III-IV GVHD compared favorably with the recent historical haploHCT cohort (2-year OS from the onset of severe GVHD was 76.2% versus 31.2% and the NRM was 14.3% versus 56.3% in the CD45RA-depleted and historic haploHCT cohorts respectively). This suggests that severe aGVHD may be relatively more treatment sensitive following CD45RA^+^depletion compared to other transplant approaches. Of note, the dose of CD45RA^+^-depleted T cells infused was approximately one log higher than that used in studies using MSD/MUD grafts.

We recently analyzed updated data from our prospective clinical trial that used CD45RA^+^-depleted haplo transplants followed by donor NK cell addback (NCT0180761) in 72 children with high-risk hematologic malignancies (46). Patients had high-risk disease, with 25 patients being in first complete remission (CR1), 28 being in second or subsequent complete remission (CR2+), and 19 having refractory disease (unable to achieve a complete remission) at the time of transplant. All patients received serotherapy-free, submyeloablative conditioning, and GVHD prophylaxis consisted of a short course of MMF or sirolimus. Patients received a CD34^+^-selected graft on day 0, followed on day +1 by a second progenitor cell graft that had been depleted of CD45RA^+^ cells. NK cells isolated from a non-mobilized apheresis product by CD3^+^ depletion/CD56^+^ selection were infused on day +6. Engraftment subsequently occurred in all but one patient, in whom a second transplant was successful. The cumulative incidence of overall aGVHD, grade III or IV aGVHD, and cGVHD was 36.1%, 29.2%, and 20.8% respectively and was higher than reported in trials in the MSD and MUD settings. The 3-year leukemia-free survival (LFS) was 88% for patients in CR1, 70.8% for patients in CR2, and 21.1% for patients with refractory disease (*P* < 0.0001). Despite the inclusion of patients with high-risk and refractory disease, the cumulative incidences of relapse and non-relapse mortality for the entire cohort were 26.5% and 11.5%, respectively. Although the rates of GVHD were higher than those observed with other T-cell–depleted grafts, this approach was associated with promising rates of LFS, relapse, and NRM in this very-high-risk patient population.

A major challenge with T-cell–depleted haplo transplants is the prolonged period of lymphopenia in the first 100 days after transplant, which places patients at increased risk of infection and relapse (50). Long-term T-cell recovery is largely dependent on *de novo* T-cell production mediated by thymopoiesis and more closely resembles that seen in age-matched controls than that in the donor graft (51). However, in the first several months post-HCT, while awaiting *de novo* T-cell production, circulating T-cells are derived directly from the donor graft. As a large number of donor T cells can be transferred with a graft that has undergone CD45RA^+^ depletion, this approach is associated with rapid T-cell reconstitution and a reduced incidence of viremia. We previously published a detailed analysis of early immune reconstitution in the first 17 CD45RA^+^-depleted haplo transplant recipients (40). An assessment of immune reconstitution in the early post-transplant period demonstrated robust reconstitution of both innate and adaptive immunity. There was rapid reconstitution of NK cells and CD8^+^ T cells to quantitatively normal levels, and a substantial number of CD4^+^ T cells were already present by day +30 (median, 160/µL). The early post-transplant T-lymphocyte subset distribution recapitulated the composition of the CD45RA^+^-depleted graft, confirming the successful adoptive transfer of balanced memory T-cell populations. Both T_EM_ cells and T_CM_ cells were present and were sustained for up to 6 months after transplantation in both the CD4^+^ and CD8^+^ T-cell populations before progenitor cell–derived thymic output. At day 100 after transplant, patients uniformly exhibited low-level TREC production, but all of them (including those with no evidence of thymic output) demonstrated substantial Vβ spectratyping diversity. These TCR data, in combination with the predominant CD45RO^+^ phenotypes, support the hypothesis that the T-cell immunity in the first months after HCT was provided by successful adoptive transfer of diverse memory T-cell populations from the CD45RA^+^-depleted grafts. In addition to the rapid phenotypic reconstitution, early functional recovery was confirmed. Proliferative responses to mitogen and antigens uniformly recovered between day +30 and day +60.

Because memory T cells retain potent virus-specific activity, we hypothesized that recipients of CD45RA^+^-depleted grafts and serotherapy-free conditioning would have better virus control when compared with recipients of CD3^+^-depleted grafts. Consistent with this hypothesis, the incidence of viremia, the peak viral loads observed, and the duration of viremia were all substantially decreased when compared with those in recipients of CD3^+^-depleted grafts. Importantly, recipients of CD45RA^+^-depleted grafts who developed viremia generally required less aggressive antiviral treatment (52).

In another study using haplo donors, Sisinni et al. reported outcomes in 25 pediatric patients who received CD45RA^+^-depleted transplants after submyeloablative conditioning (47). GVHD prophylaxis consisted mostly of a short course of CSA or MMF. Engraftment occurred in all patients, but two experienced a secondary graft rejection. The cumulative incidence of grade II-IV acute GVHD was 39% and grades III-IV acute GVHD was 33% respectively but only one patient required a second agent for treatment. The incidence of cGVHD was 22% at 30 months, and all three patients had mild or moderate disease. Importantly, no patient died of GVHD. Immune reconstitution was rapid, and although patients developed viremia, this did not progress to viral disease due to CMV, adenovirus, or EBV. However, there was an unexpectedly high rate of encephalitis due to HHV-6, with a cumulative incidence of 34% at a median of 35 days post-transplant. Patients responded to treatment, and no patient died directly of encephalitis. The cumulative incidence of relapse was 20%, and of the three patients who experienced relapse, two had active disease at the time of transplant. Overall survival was 60% and was comparable to that with transplantation from unrelated donors.

Although the results of this study are encouraging overall, the rates of HHV-6 encephalitis were concerning. In almost all patients, HHV-6 encephalitis was preceded by a pattern of pre-engraftment syndrome. In a follow-up paper, Perruccio et al. reported unusually high rates of HHV-6 reactivation and disease in two cohorts of pediatric transplant recipients in whom novel graft manipulation strategies were employed (53). The first cohort comprised 13 patients who received CD34^+^-selected transplants followed by T-Reg and T-conventional cell infusion, and the second cohort comprised 25 patients who received CD45RA^+^-depleted transplants as described above. *In vitro* experiments demonstrated that donor CD4^+^ T cells are the reservoir of HHV-6, and they suggested that the graft composition (rich in CD4^+^ T cells and poor in NK cells) contributed to the high rate of HHV-6 infections in both transplant settings. Indeed, in our institutional cohort of pediatric patients who underwent haplo transplants using CD45RA^+^ depletion followed by NK cell addback, we did not observe a high incidence of HHV-6 encephalitis. In keeping with the *in vitro* findings and encouraged by the results of our study, the same group of investigators in Spain performed a follow-up study wherein they incorporated a NK-cell infusion within 10 days after a CD45RA^+^-depleted transplant (48). The goal was to infuse a number of NK cells equivalent to at least twice the number of CD4^+^ T cells infused with the CD45RA^+^-depleted fraction, as *in vitro* work showed that NK cells could eliminate HHV-6B CD4^+^ T cells when present at a ratio of 2:1 (53). The primary endpoint in this study was the incidence of HHV-6 encephalitis. Although HHV-6B reactivation occurred in seven of the haplo HSCT recipients, it was noted only in those patients who received a cell infusion with an NK/CD4^+^ ratio of less than 2. Importantly, none of the patients developed HHV-6 encephalitis. An additional strategy to further reduce the rate of HHV6 infection could be to reduce the number of memory T cells infused. This might also reduce the rates of severe acute and chronic GVHD.

(Mercedes) Gasior Kabat and colleagues evaluated the use of CD45RA^+^-depleted transplants in five patients with bone marrow failure (four with severe aplastic anemia and one with congenital amegakaryocytic thrombocytopenia) (45). All patients received submyeloablative conditioning with TLI/Fludarabine/Cyclophosphamide. GVHD prophylaxis consisted of Cyclosporine (CSA) or MMF +/- corticosteroids. One patient received a transplant from a MMUD, and the others received transplants from haplo donors. All patients experienced rapid engraftment, and no severe infections were observed before day 100. Two patients developed grade II or higher aGVHD, and one patient developed severe cGVHD of the lung that was fatal. Two of the severe GVHD cases (grade IV acute and severe chronic) occurred in the patients who received MMF alone. There was a 60% incidence of transplant-associated microangiopathy, possibly due to prolonged CSA use. Four of 5 patients received additional CD45RA^+^-depleted DLIs: three patients for lymphopenia and viral reactivation and one patient for prophylaxis. Only 1 of 4 patients developed *de-novo* GVHD following DLI, in the other patients GVHD was not related to DLI. Three of the five patients remain alive, suggesting the feasibility of such an approach; however, further refinements are needed to improve outcomes.

Although the use of CD45RA^+^depletion has provided a therapeutic option for patients lacking matched donors, there is much work to be done to improve outcomes. Several potential strategies could be considered. Critically, the optimal dose of CD45RA^+^depleted T-cells that can safely be infused in the haplo-identical setting is yet to be determined and it is possible that lowering the dose will be associated with lower rates of GVHD. In addition, while avoiding the use of GVHD prophylaxis is the goal, a short course of immune suppression following HCT might be required to mitigate the severity of GVHD. Additional strategies might consist of co-infusing T-regulatory cell with a lower dose of CD45RA^+^depleted T-cells in an effort to tilt the balance towards lowering the risk of GVHD. Future studies must be designed to continue to interrogate these and other strategies with the goal of optimizing the therapeutic potential of CD45RA^+^-depleted haplo-identical donor grafts.

## Donor lymphocyte infusions

The adoptive transfer of donor T cells has been used to optimize immune reconstitution after transplant but is associated with an increased risk of GVHD. As CD45RA^+^-depleted T cells are associated with low rates of alloreactivity and, therefore, with low rates of GVHD but retain specificity for leukemic and viral antigens, they have been used by several investigators as donor lymphocyte infusions after transplant in an attempt to enhance immune reconstitution. Data are summarized in **Table 1B**.

**Table 1B:** Clinical trials and outcomes after CD45RA-depleted Donor Lymphocyte Infusions.

Site	Diagnosis,patient numbers	Donor type	Transplant platform	Indication, timing,cell dose used	OS, DFS	Acute GVHD	Chronic GVHD
Dunaikina et al. (54), Russia	Children with hematologic malignancies (Total = 143. experimental arm, memory DLI, n = 76; control arm, n = 73)	Haplo (n = 69) MUD (n = 6) MSD (n = 1)	TCR αβ depletion Myeloablative conditioning	Prophylactic DLI Dose 25 × 10^3^ cell/kg on day 0, then 50 ×10^3^ cell/kg on days 30, 60, 90, and 120 Median number of DLI given = 4 (range:1-5)	2-year OS: 79% (95% CI: 69%–88%) 2-year EFS: 72% (95% CI: 62%–83%)	Grade II–IV: 14.5% (95% CI: 8%–25%) Grade III or IV: 8% (95% CI: 4%–17%)	At 2 years: 6% (95% CI: 2%–16%)
Maung et al. (55), Duke (NCT01627275)	Adults with hematologic malignancies (n = 16)	MSD (n = 8) MUD (n = 8)	Reduced-intensity conditioning	Prophylactic DLI First dose given at a median of 112.5 days (range 76–280) post HCT Dose DL1: 1 × 10^5^ cells/kg DL2: 1 × 10^6^ cells/kg DL3: 5 × 10^6^ cells/kg DL4: 1 × 10^7^ cells/kg	2-year OS: 50% 2-year EFS: 68.8%	1 patient	1 patient
Naik et al. (56), St Jude (NCT03849651)	Children with acute leukemia (n = 30)	Haplo (n = 30	TCR αβ depletion Submyeloablative conditioning	Prophylactic DLI Given two weeks following engraftment Dose DL1: 1 × 10^5^ cells/kg DL2: 1 × 10^6^ cells/kg DL3: 1 × 10^7^ cells/kg	1-year OS: 86.3% (95% CI: 74.6%–99.7%) 1-year EFS: 69.8% (95% CI: 55.2%–88.4%)	Grade II–IV: 26.7% (95% CI: 2.4%–43.3%) Grade III or IV: 13.3% (95% CI: 4.1%–28.1%)	None
Castagna et al. (57), Italy (NCT04687982)	Adults with hematologic malignancies (n = 19)	Haplo (n = 30)	Post-transplant cyclophosphamide Myeloablative, reduced-intensity conditioning	Prophylactic DLI of 3 infusions each 4-6 weeks apart First dose given at median of 55 days (range, 46-63) post HCT Dose 5 × 10^5^ cells/kg 1 × 10^6^ cells/kg 5 × 10^6^ cells/kg	1-year OS: 79% (95% CI: 45%–93%) 1-year EFS: 75% (95% CI: 46%–90%)	aGVHD: 6% (95% CI: 0%–17%)	At 1 year: 15% (95% CI: 0%–35%).
Muffly et al. (58), Stanford (NCT01523223)	Adults with relapsed hematological malignancies (n = 15)	MSD (n = 15)		DLI for relapse Median time from disease relapse to DLI: 236 days (range, 7-3005 days) Dose 1 × 10^6^ cells/kg 5 × 10^6^ cells/kg 10 × 10^6^ cells/kg	Median of 328 days post infusion Median OS: 19.6 months (95% CI: 5.6 months to not reached) Median EFS: 4.9 months (95% CI: 1–19.3 months)	1 patient	

DLI, donor lymphocyte infusion; DL, dose level; HCT, hematopoietic cell transplant; MSD, matched sibling donor; MUD, matched unrelated donor; MMUD, mismatched unrelated donor; Haplo, haploidentical donor; GVHD, graft-versus-host disease; OS, overall survival; EFS, event-free survival.

## Enhancement of immune reconstitution

Dunaikina and colleagues evaluated the safety and efficacy of low-dose memory (CD45RA^+^-depleted) donor lymphocyte infusion (mDLI) given early after HCT with TCRαβ T-cell–depleted grafts (54). The goal of limiting the dose of mDLI to was to prevent GVHD, and early application was projected to prevent the reactivation of key viral pathogens. A cohort of 149 children was enrolled, of whom 76 were randomized to receive scheduled mDLI and 73 to receive standard care. The incidences of grade II–IV aGVHD were comparable in the experimental and control arms (*P* = 0.8). The rates of OS, EFS, NRM, and relapse were similar for the two study arms, demonstrating the safety of this approach. The incidence of CMV viremia, which was the endpoint used to define efficacy in this study, was comparable in the two arms, being 45% in the experimental arm and 55% in the control arm (*P* = 0.4). However, mDLI was associated with improved recovery of CMV-specific T-cell responses in a subcohort of CMV IgG–seropositive recipients, highlighting the key role of antigen exposure in the expansion and persistence of virus-specific T cells derived from mDLI.

Maung and colleagues assessed the safety of CD45RA^+^-depleted T-cell infusion to augment post-transplant immune recovery without increasing the risk of GVHD (55). The transplant regimen consisted of non-myeloablative conditioning therapy that incorporated serotherapy (either ATG or Alemtuzumab) followed by a T-replete HLA-identical graft. Sixteen adult patients received escalating doses of prophylactic CD45RA^+^-depleted DLI at a median of 113 days (range, 76–280 days) after transplant. No dose-limiting grade III or IV aGVHD was observed, suggesting again that prophylactic mDLI is safe and is not associated with high rates of aGVHD or cGHVD. The sample size was too small to draw conclusions regarding the GVL effect or the impact on functional immunologic recovery

At our institution, we implemented a prospective trial (NCT03849651) using escalating doses of CD45RA^+^-depleted T cells as addback after TCRαβ-depleted haplo transplant to enhance immune reconstitution in pediatric patients with high-risk acute leukemia (56). We performed an interim analysis of the first 30 patients. Two weeks after engraftment, patients received CD45RA^+-^depleted T-cell addback in three escalating doses. At 1 month post infusion, there was a significant increase in the median number of CD3^+^ T cells, including CD8^+^ and CD45RO^+^ T-cell subsets (*P* < 0.01, *P* < 0.001). There was also significant expansion of virus-specific T cells (VSTs) directed towards CMV, adenovirus, BK virus, or HHV-6, as shown by enzyme-linked immune absorbent spot (ELISpot) assays (*P* < 0.01). The TCR repertoire, as assessed by Vβ-spectratyping, was broad and comparable to that of the donor by month 6 post-transplant. The incidences of aGVHD within 28 days after infusion of CD45RA^+^-depleted T cells at dose levels 1, 2, and 3 were 0%, 20%, and 10%, respectively (the differences were not statistically significant), suggesting that the infusion of T-cell doses as high as 1 × 10^7^ T-cells/kg was safe. There was no chronic GVHD; however, the follow-up was short. This interim analysis suggests that the approach is safe at the dose levels used without high rates of concurrent GVHD. Comparative studies will be needed to assess the impact of CD45RA^+^-depleted T cells to enhance immune reconstitution.

Recently, Castagna et al. evaluated the role of CD45RA^+^-depleted DLI after haplo transplantation with post-transplant cyclophosphamide for patients with hematologic malignancies (57). CD45RA^+^-depleted DLI was delivered in three escalating doses, 4–6 weeks apart, with the first infusion being delivered at a median of 55 days after transplant. Sixteen of the 19 patients received all three doses. Infusion proved to be safe and well tolerated, and there was no increase in the rate of aGVHD or cGVHD. The primary endpoint was the rate of viral infections at day 100. Landmark analysis was performed to take into account the timing of CD45RA^+^-depleted DLI. Compared with previously published data from this group, the incidence of viral reactivation was reduced from 53% (95% CI, 41%–66%) to 32% (95% CI, 10%–53%). While these findings are promising, there are inherent limitations of landmark comparison with retrospective control groups. Future strategies could include randomized control trials to determine whether CD45RA^+^-depleted DLIs can indeed lower infectious complications.

## Treatment of relapse

Muffly and colleagues (58) evaluated the feasibility and safety of infusing freshly isolated and purified donor-derived phenotypic CD8^+^ memory T cells into adults with relapsed disease after allogeneic HCT (NCT01523223). This was based on preclinical work showing that CD8^+^ memory T cells, but not CD4^+^ memory T cells, mediated potent antitumor effects and could eradicate tumors in mouse models as effectively as could unmanipulated DLIs but without causing GVHD. CD8^+^ T cells were isolated after non-mobilized donor apheresis by using a tandem immunomagnetic selection strategy of CD45RA^+^ depletion followed by CD8^+^ enrichment. Fifteen patients received CD8^+^ memory T cells at escalating doses. Ten of these 15 patients (67%), of whom four had active disease at the time of infusion, responded to the treatment (seven had complete remission, one had partial remission, and two had stable disease) for at least 3 months after infusion. Five patients did not respond (all had active disease at time of infusion). Of the nine patients with active disease at the time of infusion of CD8+CD45RA-depleted DLI, five had progressive disease at 3 months, two had stable disease at 3 months and progressive disease by six months, one had a partial response at 3 months and progressive disease by six months, and one had a durable CR. Only one patient developed GVHD (grade 2 liver disease that was steroid responsive), thereby confirming the preclinical findings and highlighting the safety of this approach as compared with conventional DLI.

## TCRαβ+ T cell-depletion

The other major *ex vivo* T cell depletion strategy currently undergoing clinical evaluation is the use of TCRαβ+ T cell-depletion. While not the primary focus of this review, we briefly discuss here the development and use of this approach as it has several important conceptual and practical similarities to naïve T cell depletion through the targeting of CD45RA.

T cells can broadly be divided into TCRαβ+ T cells, that represent 80-90% of circulating T cells and, TCRγδ+ T cells that comprise about 1-20% of circulating T cells but are the majority of resident T cells in the skin and mucosa (59).Studies have shown that TCRαβ+ T cells play a more central role than TCRγδ+ populations in causing GvHD (60–62). Pre-clinical studies using mice transplanted with MHC-incompatible marrow grafts demonstrated that when infused with large doses of *ex vivo* activated and expanded TCRγδ+ T cells, mice exhibited successful engraftment but showed no evidence of GVHD (63). In addition, TCRγδ+ T cells can mediate innate immunity against a wide variety of infections, play a role in pathogen clearance and have anti-tumor effects with lytic activities demonstrated against hematological malignancies and solid tumors (64–67). It was therefore hypothesized that selective depletion of TCRαβ+ T cells and the preservation of TCRγδ+ T cells in the graft along with NK cells and other cell populations might offer an additional advantage in a clinical transplant setting.

Chaleff et al. developed a technique to perform clinical-scale TCRαβ+/CD19+ depletion of GCSF mobilized PBSCs using the CliniMACS device (68). The two-step method resulted in a 4-5 log depletion of TCRαβ+ T cells while retaining TCRγδ+ T cells and NK cells. Subsequent reports confirmed efficacy of this approach and demonstrated > 70% recovery of CD34+ cells (69).

Bertaina et al, were among the first to report results from a clinical trial using TCRαβ+ T cells along with CD19+ depletion of haplo grafts in 23 children transplanted for non-malignant disorders (70). The median number of CD34, TCRαβ+ T cells, TCRγδ+ T cells and B cells infused was 15.8 x10^6^cells/kg, 40 x10^3^ cells/kg, 9.4 x10^6^cells/kg and 40 x 10^3^ cells/kg, respectively. Patients received disease-specific conditioning regimens that incorporated ATG and Rituximab. No post-HCT GVHD prophylaxis was used. Only 3 of 23 patients developed grade I - II acute skin GVHD. Impressively, no patient developed visceral GVHD or any Grade II-IV GVHD. At a median follow-up of 18 months, the 2-year probability of disease-free survival was 91.1% The same group reported results from a cohort of 80 patients with acute leukemias (71) transplanted with TCRαβ+/CD19+ depleted haplo grafts. Patients received a fully myeloablative preparative regimen with ATG and no post-HCT GVHD prophylaxis was used. Here again patients developed only grade I - II acute skin GVHD (cumulative incidence of 30%) and no patient developed any grade visceral or extensive cGVHD. The cumulative incidence of relapse was 24% and with a median follow-up of 46 months the 5-year probability of GRFS was 71%. The outcomes of these 80 patients were comparable to those of 41 and 51 children who received T cell-replete transplants from an MSD or a MUD respectively in the same period.

Multicenter studies have confirmed these single center results. In a large Italian multi-center study, outcomes of TCRαβ+/CD19+ depletion using haplo donors (n=98) were compared with T cell-replete transplants from MUD (n= 127) or MMUD (n= 118) in pediatric patients with acute leukemias (72). Again, there were no cases of grade III–IV skin or any visceral aGVHD in recipients of TCRαβ+/CD19+ depleted haplo donor grafts. Incidence of grade III–IV aGVHD and extensive cGVHD were significantly lower than those seen in MUD or MMUD. LFS was comparable between recipients of TCRαβ+/CD19+ depleted haplo HCT and recipients of T cell-replete MUD and MMUD HCT (65%, 55%, and 62% respectively). While comparable to T cell-replete MUD, the GRFS of TCRαβ+/CD19+ depleted haplo HCT recipients was superior to MMUD recipients as was the NRM, suggesting that using this strategy, haplo donors could be an effective donor choice, especially when a MUD was not available. Lang et al. also reported on a multicenter phase I/II trial of transplantation of TCRαβ+/CD19+ depleted PBSCs from haploidentical family donors but using a reduced-intensity conditioning regimen with fludarabine, thiotepa, and melphalan (73). ATG was incorporated in the conditioning regimen and GVHD prophylaxis consisted of mycophenolate mofetil. Thirty pediatric and adult patients with malignant and non-malignant diagnoses were transplanted. Patients not in CR (n=18) were also included. No patients developed grade III-IV GVHD. The overall and disease-free survival at 2 years were 63% and 50%, confirming this approach was feasible in high-risk patients using RIC regimens.

Based on the success of these studies using haplo-identical donors, the use of TCRαβ+ T cell depletion has been extended to the unrelated donor setting. Leahy et al. reported on a multi-center study of sixty pediatric and young adult patients with hematologic malignancies who lacked a matched-related donor (74). All patients received myeloablative total body irradiation– or busulfan-based conditioning followed by TCRαβ+ T cell depleted graft from a MUD. Patients did not receive posttransplant immune suppression and only 1/3^rd^ received serotherapy with ATG. Four-year OS was 69% and LFS was 64%. Three-year cumulative incidence of relapse and NRM was 21% and 15% respectively. Despite the lack post-transplant immune suppression and avoidance of ATG in 2/3^rd^ of recipients, grade III to IV aGVHD was seen in only 13% of patients and 11% developed extensive cGVHD. Nonpermissive DP mismatch was associated with higher likelihood of aGVHD but not with the development of cGVHD. Overall, these data demonstrate that suggested that TCRαβ+/CD19+ depleted MUD transplant was a safe and effective approach to transplantation for patients with leukemia.

Maschan et al. evaluated the use of TCRαβ+/CD19+ depleted grafts using MUD and mismatched related donors (MMRDs) in patients with primary immunodeficiency (PID) (75). Outcomes were compared amongst a total of 98 pediatric patients with various PIDs transplanted using MUDs (n = 75) or MMRDs (n = 23). Conditioning was based on a fludarabine-/treosulfan-backbone and majority (n= 73) also receiving a second alkylating agent. All but 2 patients received serotherapy (ATG) before HCT and a short course of posttransplant immune suppression. There was no difference in the cumulative incidence of aGVHD grade 2 to 4 at 17% in the MUD group and 22% in the MMRD group (*P* = .7). The OS was 86% in the MUD group and 87% in the MMRD group (*P* = .95) suggesting there was no difference in outcomes of HCT performed from MUD and MMRD using this approach for patients with PID.

Several groups have since reported on successful use of TCRαβ+/CD19+ depleted transplant in various settings including for non-malignant diseases (76) including PIDs and Severe Combined Immune Deficiency (SCID) (77), Fanconi Anemia (78), and Sickle Cell Disease (79). Other trials have also confirmed efficacy in patients with malignancies (80, 81).

These promising results have led to the adoption of more widespread use of TCRαβ+ T cell depletion. In general, there is more published data available in the pediatric setting using the TCRαβ+ T cell depletion approach and with the use of haplo-identical donors where very low rates of grade III-IV GVHD have been reported. In contrast, the naive T cell depletion platform has been employed more frequently in adults in the in the MSD and MUD setting. While data for haplo trials exists using naïve T-cell depletion, the rates of GVHD appear higher than those reported using TCRαβ+ T cell depleted haplo grafts, perhaps due to the higher number of T cells infused. The naïve depleted graft contains more T-cells, specifically memory T-cells, than the TCRαβ+ T cell depleted graft. This could theoretically translate into a) improved immune reconstitution, reduced opportunistic infections and infectious deaths and b) possibly an improved GVL effect. However, these advantages remain to be proven in prospective studies. Nevertheless, the use of TCRαβ+ T cell depletion and naïve T cell depletion have advanced the field of transplantation and serve as excellent platforms to enhance the safety and efficacy of allogeneic transplantation. At this time, the use of either approach is based on center experience and institutional preference, and there are currently no randomized trials comparing these techniques. Future trials comparing the approaches should evaluate key variables, besides GVHD, such as relapse and NRM with the goal to provide further insight into advantages and disadvantages of each approach. As described above, investigators are also combining these two approaches by using TCRαβ+/CD19+ depleted progenitor cell grafts and naïve T-cell depleted DLI in an attempt to incorporate the advantages of each strategy into one transplant platform.

## Conclusions and future directions

With more advanced graft engineering techniques and the development of “designer grafts” that can enhance immune reconstitution and at least partially separate the GVL effect from GVHD, there is potential for much broader use of CD45RA^+^ depletion. Data from ongoing and completed clinical studies detailed in this review highlight the safety of infusing CD45RA^+^-depleted T cells and demonstrate the association of this approach with rapid and robust immune reconstitution and reduced viral disease. Future steps include optimizing the number of CD45RA^–^ T cells that can be infused, particularly in the haplo donor setting, and determining the most optimal conditioning regimens and GVHD prophylaxis to maximize the benefit of these cells. Lastly, although this approach has been more extensively studied in patients with leukemias, it could be extended to patients undergoing HCT for nonmalignant conditions.

## Author contributions

All authors contributed to the article and approved the submitted version.
